# Androgen deprivation therapy plus apalutamide as neoadjuvant therapy prior radical prostatectomy for patients with unresectable prostate cancer

**DOI:** 10.3389/fphar.2023.1284899

**Published:** 2023-10-19

**Authors:** Yongbao Wei, Ruochen Zhang, Dewen Zhong, Zhensheng Chen, Gen Chen, Minggen Yang, Le Lin, Tao Li, Liefu Ye, Lili Chen, Qingguo Zhu

**Affiliations:** ^1^ Shengli Clinical Medical College of Fujian Medical University, Fuzhou, China; ^2^ Department of Urology, Fujian Provincial Hospital, Fuzhou, China; ^3^ Department of Urology, Longyan First Affiliated Hospital of Fujian Medical University, Longyan, Fujian, China; ^4^ Department of Urology, Fuding Hospital Affiliated to Fujian University of Traditional Chinese Medicine, Ningde, Fujian, China; ^5^ Department of Urology, Gutian County Hospital, Ningde, China; ^6^ Department of Urology, Zhangzhou Affiliated Hospital of Fujian Medical University, Zhangzhou, China; ^7^ The School of Nursing, Fujian Medical University, Fuzhou, China

**Keywords:** prostate cancer, androgen deprivation therapy, apalutamide, neoadjuvant therapy, radical prostatectomy

## Abstract

Whether neoadjuvant therapy confers a survival benefit in advanced prostate cancer (PCa) remains uncertain. The primary endpoints of previous retrospective and phase II clinical studies that used neoadjuvant therapy, including androgen deprivation therapy combined with new-generation androgen receptor signaling inhibitors or chemotherapy, were pathological downstaging, progression-free survival, prostate-specific antigen relief, and local symptom improvement. To the best of our knowledge, no studies have explored the efficacy and safety of neoadjuvant therapy in improving the surgical resection rate in cases of unresectable primary tumors of PCa. We first designed this retrospective study to evaluate the potential value of apalutamide as neoadjuvant therapy in improving the resectability rate of radical prostatectomy (RP). We initially reported 7 patients with unresectable primary lesions who underwent neoadjuvant apalutamide treatment for a median of 4 months, and all of them successfully underwent RP treatment. Our study supported apalutamide as neoadjuvant therapy, which helped improve RP’s success rate and did not significantly increase perioperative complications, and the neoadjuvant therapy was controllable. Our findings’ clinical value and benefit for survival still need further clinical research to confirm.

## Introduction

The main goals of neoadjuvant therapy for prostate cancer (PCa) based on androgen deprivation therapy (ADT) are to reduce tumor size, reduce the rate of positive surgical margins, and achieve pathological remission, but no benefit has been observed in terms of cancer-related death (Ge et al., 2022; Wang et al., 2022). Thus, neoadjuvant therapy has not been recommended as the primary treatment option for patients with progressive PCa (Ge et al., 2022; Wang et al., 2022). In the past 20 years, the emergence of new-generation androgen receptor signaling inhibitors (ARSI), such as abiraterone, apalutamide, enzalutamide, and darolutamide, has increased interest in the neoadjuvant treatment of PCa (Devos et al., 2021). Recently, some phase II studies of apalutamide as neoadjuvant therapy, mainly for newly diagnosed patients with medium and high-risk PCa, including a single-arm phase II NEAR trial (Lee et al., 2022; Yang et al., 2022) and a placebo-controlled phase II study (Devos et al., 2023), both their primary endpoints were pathological response rate and safety. Another phase II study explored the effect of apalutamide as neoadjuvant therapy on perioperative complications and found that it did not significantly increase the occurrence of significant complications of grade 3 and above; however, it might increase the risk of thrombosis in patients with RP and lymph node dissection (Ilario et al., 2023).

There is no phase Ⅲ clinical study data to prove the value of survival benefit in neoadjuvant therapy for progressive PCa. Several phase II clinical studies of ARSI and chemotherapy as neoadjuvant therapy, including docetaxel (Zhuang et al., 2023) or cabazitaxel (Fleshner et al., 2023), main focus is on pathological response rate, pathological downstaging rate, prostate-specific antigen (PSA) benefit, progression-free survival (PFS) and complications. There is no report on the efficacy and safety of neoadjuvant therapy for unresectable progressive PCa. We designed this retrospective study to evaluate the potential value of apalutamide as neoadjuvant therapy in improving the resectability rate of the prostate.

## Methods

**FIGURE 1:**
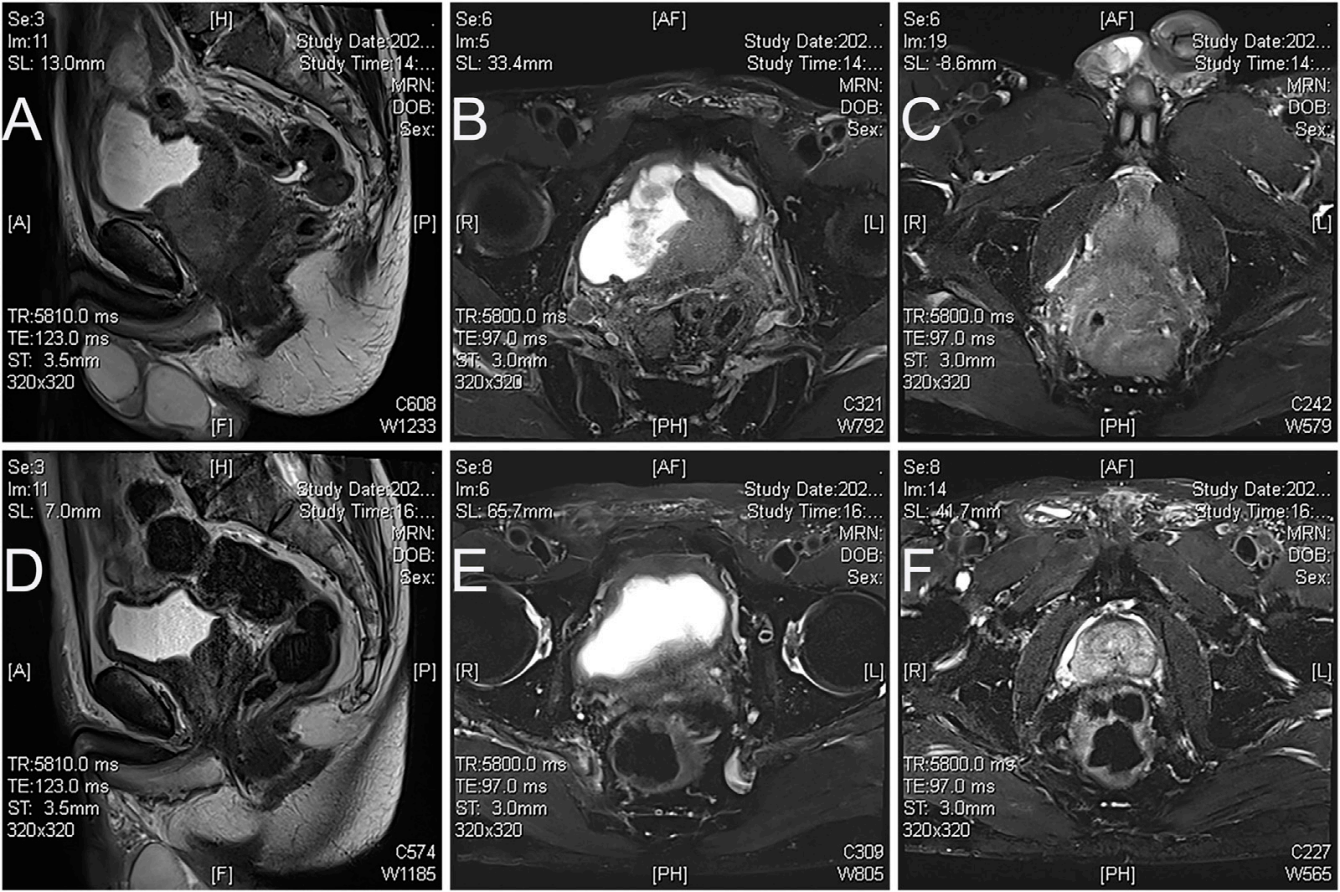
Magnetic resonance images of apalutamide before and 3 months post neoadjuvant treatment in case 2. **(A)**, sagittal view, (**B,C)**, axial view T2 imaging showed that prostate cancer broke through the prostate capsule and invaded the rectum, bladder neck, and seminal vesicles before neoadjuvant treatment; **(A)**, sagittal view, **(B,C)**, axial view T2 imaging showed that prostate cancer retracted significantly after neoadjuvant therapy **(D)**, the boundary between the prostate and rectum seemed more straightforward, with no apparent tumor involvement in the bladder neck, but lesions invaded seminal vesicle still exists **(E,F)**.

We included PCa patients diagnosed and treated by the Fujian Prostate Disease Diagnosis and Treatment Alliance (including 45 medical centers) from January 2021 to August 2023. Patients were screened according to the following inclusion and exclusion criteria. The inclusion criteria were as followings: (a) the patient was diagnosed with advanced prostate acinar adenocarcinoma (T4N0-1M0-1); (b) their physical status score were 0 according to Eastern Cooperative Oncology Group (ECOG) (Anker et al., 2016); (c) the patient had completed the multi-parameter magnetic resonance imaging (MRI) plain scan and enhanced examination for prostate before the prostate biopsy (Figure 1), and evaluated by 2 PCa surgeons from Fujian Provincial Hospital as unresectable PCa with progressive primary prostate lesions (defined as PCa with local progression and mainly invading the rectum, bladder and surrounding tissues, and the primary prostate lesions and seminal vesicles could not be removed without avoiding cystectomy or rectal injury; each surgeon had completed 100 or more PCa laparoscopic or robotic surgeries in the past 2 years); (d) patients had undergone at least 3 months of ADT combined with apalutamide 240 mg once daily as neoadjuvant therapy; (e) prostate MRI was re-evaluated after neoadjuvant treatment to evaluate whether the prostate resectable; (f) critical data such as imaging and PSA re-examination were available and the patients were willing to participate this study. Exclusion criteria were as follows: (a) patients with poor physical status (ECOG ≥1); (b) the evaluation of the prostate by MRI or 99mTc-prostate-specific membrane antigen Computed Tomography (PSMA CT) (Zhang et al., 2022) after neoadjuvant treatment suggested no tumor activity in the prostate; (c) patients with more than 5 bone metastatic foci or combined with visceral metastase (not an oligometastasis) (Wenzel et al., 2023); (d) patients who were not suitable for surgery or who had limited benefit from surgical treatment evaluated by consultation from PCa multidisciplinary team (MDT). The primary endpoint of our study was the number of patients with resectable surgery after neoadjuvant apalutamide therapy. The secondary endpoints were the number of tumor stage remission, the number of pathological remission, and the number of PSA relief (defined as PSA less than 0.10 ng/mL from the start of apalutamide treatment to half a year after surgery), as well as perioperative complications and drug side effects of neoadjuvant treatment.

## Results

**FIGURE 2:**
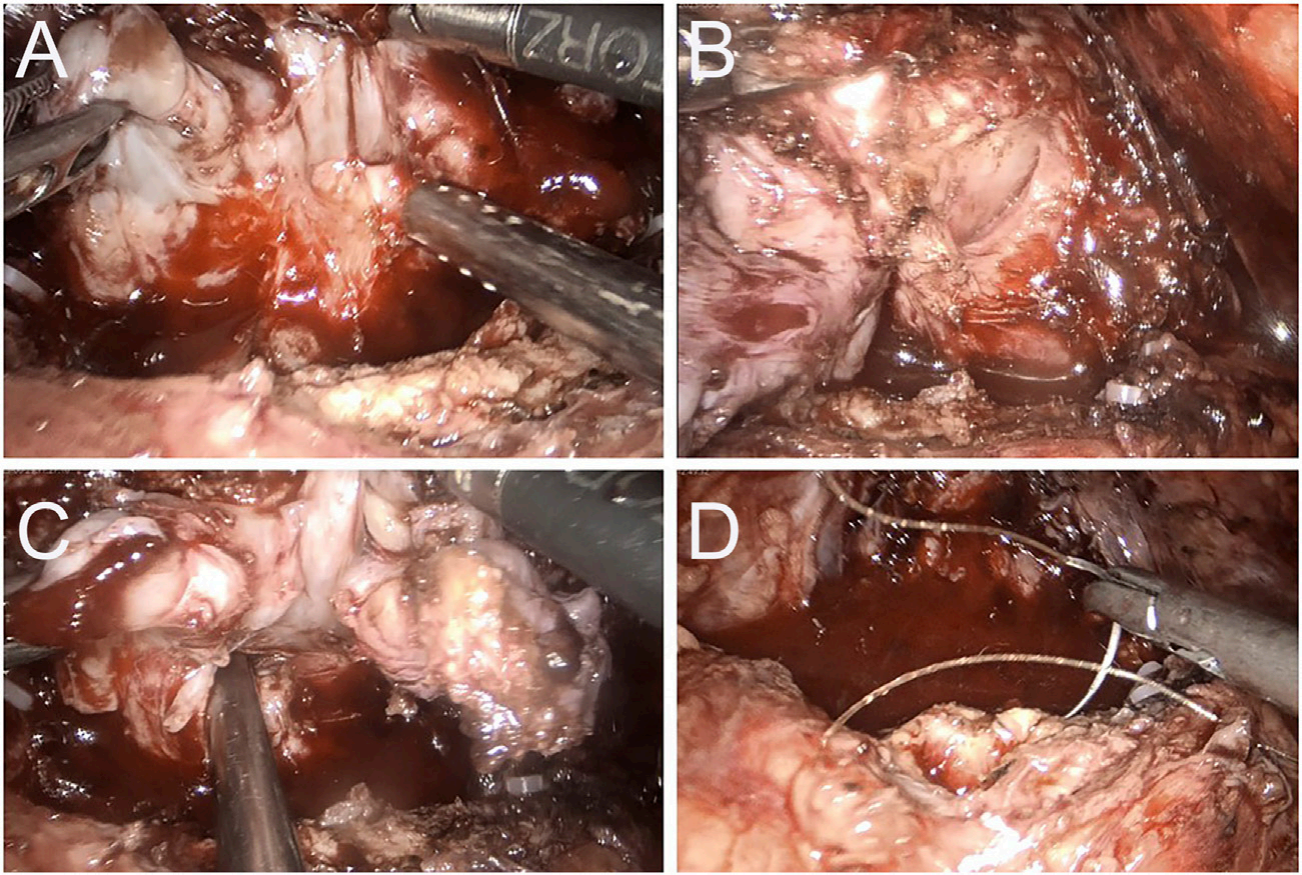
Case 2 Intraoperative image of 3 months after apalutamide neoadjuvant therapy. **(A)** The seminal vesicles on both sides are still evident, but apparent adhesions to the rectum are observed; **(B)** a clear separation between the right lobe of the prostate and the rectum; **(C)** the adhesion band with the rectum at the angle of the left seminal vesicles; **(D)** no apparent tumor invasion on the bladder neck and prostate is completely removed.

We finally included 7 patients (Table 1). Their median age was 67 years old (range 65–81 years old), with a median Body mass index of 23.8 kg/m^2^ (range 21.7–26.3 kg/m^2^). Their median initial PSA was 70.12 ng/mL (range 17.34–271.58 ng/mL), and their median needle biopsy Gleason score was 9 points (range 8–10 points). Their newly diagnosed stage was cT4N0-1M0-1b. Their median ADT plus apalutamide treatment duration was 4 months (range 3–12 months). Preoperative PSA was less than 0.01–4.09 ng/mL. After neoadjuvant treatment, these patients were all re-evaluated as prostate resectable, and all underwent RP or cytoreductive proctectomy (Figure 2), with standard or extended lymph node dissection simultaneously. Their median operative time was 200 min (range 140–300 min), and the median intraoperative blood loss was 60 mL (range 50–120 mL). All patients showed improved lower urinary tract symptoms and downgraded Gleason score after radical prostatectomy, with 1 case achieving pathological complete remission. A decrease in PSA was observed at 1.5 months after surgery, and 5 cases had PSA relief. The median follow-up time was 13 months (range 9–25 months). By the end of the follow-up, 6 cases had sustained PSA relief, and 1 case had PSA decline and then increase, which may be related to the progression of metastatic lesions. No symptom progression was observed in all patients. Neoadjuvant therapy did not significantly increase perioperative complications; no grade 3 or above complications were found (Dindo et al., 2004). Urinary incontinence over 2 months was not observed. No new adverse reactions from neoadjuvant therapy were observed, and the side effects were tolerable.

**TABLE 1:** Clinicopathological details for this case series.

Cases^a^	Age (years)	BMI (kg/m^2^)	Initial PSA (ng/mL)	Needle biopsy Gleason score	cTNM stage	Previous treatments	Pre-surgery PSA (ng/mL)	Duration of surgery (minutes)	Intraoperative blood loss (mL)	Radical prostatectomy Gleason score	ypTNM	Post-surgery PSA (ng/mL)	Post-surgery treatments	PSA (ng/mL) at last follow-up	Follow-up (months)	Surgical complications and grading	\Medication treatment-related adverse events
1	65	23.8	34.23	4 + 4 = 8	T4N1M0	Apalutamide (3 months)	<0.01	190	50	pathological complete response	T0N0M0	<0.01	Apalutamide	<0.01	18	Incontinence for 1 month, Grade 1	Hypertension Grade 1
2	67	26.2	88.49	5 + 5 = 10	T4N1M0	Apalutamide (3 months)	0.78	240	80	5 + 4 = 9	T3bN0M0	<0.01	Apalutamide	<0.01	11	Incontinence for 2 weeks, Grade Ⅰ	Decreased appetite Grade Ⅰ; Rash, Grade Ⅱ; (recovery after reduction)
3	67	21.7	17.34	4 + 5 = 9	T4N0M0	Apalutamide (3 months)	<0.01	230	50	4 + 4 = 8	T2N0M0	<0.01	Apalutamide	<0.01	10	Incontinence for 1 month, Grade Ⅰ	Fatigue, Grade Ⅰ
4	81	22.4	271.58	5 + 4 = 9	T4N1M1b	Apalutamide (12 months)	4.09	140	50	4 + 3 = 7	T3bN0M1b	1.32	Apalutamide; Abiraterone plus Olaparib	7.93	20	Incontinence for 3 weeks, Grade Ⅰ	Rash Grade Ⅱ (recovery after reduction)
5	66	25.6	41.81	5 + 4 = 9	T4N1M1a	Apalutamide (6 months)	1.34	300	120	4 + 3 = 9	T3aN1M1a	0.10	Apalutamide	<0.01	9	Incontinence for 1 month, Grade Ⅱ	Pruritus, Grade Ⅰ
6	74	26.3	70.12	4 + 4 = 8	T4N0M0	Apalutamide (4 months)	<0.01	150	100	3 + 4 = 7	T2N0M0	<0.01	Apalutamide	<0.01	25	Incontinence for 2 weeks, Grade Ⅰ	Anaemia, Grade Ⅰ; Hypertriglyceridemia, Grade Ⅰ
7	67	23.5	147.85	5 + 5 = 10	T4N1M1b	Apalutamide (4 months)	0.09	200	60	4 + 4 = 8	T2N0M1b	<0.01	Apalutamide	<0.01	13	Incontinence for 3 weeks, Grade Ⅰ	Not observed

^a^ Androgen deprivation therapy is the baseline treatment for these patients; BMI, body mass index, weight (in kg)/height^2 (in m^2); cTNM, clinical TNM; TNM, according to Union for International Cancer Control TNM, classification of malignant tumours, 8thedition; ypTNM = TNM, staging after neoadjuvant therapy; PSA, prostate-specific antigen.

## Discussion

To date, we first reported the potential value of neoadjuvant therapy for unresectable progressive PCa and neoadjuvant therapy did not significantly increase perioperative complications, and the safety was tolerable.

In recent years, some phase II studies of apalutamide as neoadjuvant therapy were performed, mainly for newly diagnosed high-risk PCa patients, with primary endpoints as pathological remission and safety. A single-arm phase II study (NEAR trial) investigating neoadjuvant apalutamide monotherapy and RP in newly diagnosed intermediate- and high-risk PCa patients, cancer burden reduction and PSA relief were obtained without any pathologic complete response (Lee et al., 2022). Neoadjuvant apalutamide treatment was tolerable and did not have a clinically significant negative impact on the patient’s overall health status and quality of life scores, with the main side effects of fatigue and sexual dysfunction (Yang et al., 2022). This study was consistent with the secondary findings of our study, but we did not assess sexual dysfunction because these patients had little need for sex before neoadjuvant therapy. In another randomized, placebo-controlled phase II neoadjuvant trial of neoadjuvant degarelix plus apalutamide compared with degarelix before RP for 12 weeks in patients with high-risk PCa, degarelix plus apalutamide significantly improveed pathological response (including minimal residual disease and residual cancer burden at final pathology) (Devos et al., 2023). From another prospective single-center phase II trial of patients with high-risk PCa, ADT plus abiraterone with or without apalutamide were given as neoadjuvant therapy and no significant difference in complications within 30 days were observed between the two arms, but 4.9% of thromboembolic events occurred in the arm of neoadjuvant triple therapy (Ilario et al., 2023). None of these studies addressed the effect of apalutamide on prostate resectability in patients with progressive PCa. In our limited cases, in addition to finding that neoadjuvant therapy helped improve prostate resectability, pathological downstaging, and PSA relief were also observed. Regarding complications, we did not perform triple neoadjuvant therapy or observe that apalutamide increased perioperative complications, and no thrombotic events were observed.

Although whether ADT combined with ARSI as neoadjuvant therapy can bring survival benefits is worthy of further research, it is relatively sure that ADT alone before RP is not recommended, especially for patients with intermediate- and low-risk PCa, as ADT can cause side effects such as weight gain and mood changes, and increases the risk of cardiovascular disease, diabetes, and osteoporosis, guidelines also strongly recommend that men who choose surgery should not undergo ADT (MacLennan et al., 2023). However, a systematic review suggested a survival benefit with ADT as a neoadjuvant approach in high-risk PCa patients (Cartes et al., 2023). For metastatic PCa (mPCa), the current mainstream view is that local treatment is not recommended (Cornford et al., 2021). However, accumulating retrospective studies have found that for selective metastatic PCa, local treatment such as RP would improve symptoms, PFS and PSA benefits, and even survival benefits (Rajwa et al., 2023). Therefore, how to select appropriate progressive PCa patients for neoadjuvant therapy and then give RP is worthy of attention. A study found that the overall mortality of mPCa benefited from local treatment, and patients with less aggressive tumors and good general health appeared to benefit more (Loppenberg et al., 2017). In our study, 4 cases were localized PCa, and 3 were mPCa. These 3 patients we included were oligometastasis and in good physical condition. After neoadjuvant therapy, RP or cytoreductive prostatectomy brought symptom improvement, PSA relief, and pathological response. However, whether this kind of benefit would bring PFS and survival benefits deserves further observation.

Evaluating which patients are suitable for RP after neoadjuvant therapy is also worth exploring. Recently, it was found that the response of prostate-specific membrane antigen (PSMA) positron emission tomography (PET)/CT to primary PCa lesions after neoadjuvant therapy could predict the pathological response, which might be helpful for the selection of patients to perform RP. In a phase II clinical trial of high-risk PCa patients receiving ADT plus docetaxel or ADT plus abiraterone as neoadjuvant therapy, with a median follow-up time of 30 months, the study found that [68 Ga] PSMA PET/CT was an ideal tool for monitoring response to neoadjuvant therapy (Chen et al., 2023). After apalutamide treatment, we selected patients who underwent MRI or PSMA CT to determine that the prostate still had active cancer lesions and then underwent RP. Another study predicted the clinical parameters and molecular biomarkers after PCa neoadjuvant chemohormonal therapy to evaluate the pathological response to neoadjuvant therapy; they found a lower preoperative PSA level was an independent predictor of good pathological response (Fan et al., 2023). The preoperative PSA levels of the patients included in our study were all low. Only one case with mPCa had a preoperative PSA level exceeding 4 ng/mL. Longer-term PSA relief postoperatively suggested that these patients may have potential survival benefits from neoadjuvant therapy; however, whether these short-term benefits could be translated into long-term survival benefits and how safe this neoadjuvant therapy was still worthy of further exploration.

How to choose follow-up therapy after neoadjuvant therapy with RP is also an uncertain issue (Devos et al., 2021). An advanced PCa consensus conference may complement the knowledge gap in advanced PCa and help the MDT discuss treatment options (Gillessen et al., 2022). It is worth noting that most of the patients selected for neoadjuvant therapy are high-risk or very high-risk PCa, multi-modal active treatment is still required after RP to improve the clinical outcomes of these patients (Devos et al., 2021; Sugino et al., 2023). Although all 7 patients in our study achieved postoperative downstaging and extremely low PSA, most were high-risk or very high-risk PCa, and active continuous multimodal treatment after surgery was also applied.

As an exploratory retrospective study with a small sample size, this study has some limitations specific to retrospective studies. In the future, case-control studies with a large sample size are needed to evaluate this study’s conclusions further. It was important to note that the patients we selected had unresectable PCa. The definition of unresectable PCa was subjective, and whether it was resectable had a close relationship with the surgeon’s surgical skills. However, our cases were all evaluated by MRI, which objectively confirmed that PCa had progressed and invaded the rectum, bladder, or surrounding tissues. In addition, our case was based on two experienced surgeons who had undergone more than 100 PCa cases, suggesting these two surgeons were already qualified in RP. After a median of 4 months of neoadjuvant therapy with apalutamide, these patients underwent MRI re-examinations, the prostate was resectable was re-evaluated, and the potential benefits of RP through MDT were also discussed to maintain as much as possible the safety of RP and the reduction of surgical complications. Furthermore, although our study had a median follow-up time of 13 months (range 9–25 months), this follow-up time was far from enough to observe patients with PCa to determine whether apalutamide as a neoadjuvant treatment brought benefit in progression-free survival, overall survival, or its long-term side effects. However, for the purpose of our research, neoadjuvant treatment with apalutamide could help transform these selective PCa patients from unresectable primary lesions to resectable primary lesions. This was the paramount significance of this preliminary study.

## Conclusion

To the best of our knowledge, we have performed the first preliminary assessment of unresectable progressive prostate cancer; those after neoadjuvant apalutamide therapy were converted to resectable prostate cancer, with no significant increase in perioperative complications and a tolerable safety of the neoadjuvant therapy. Our study provides a new opportunity for RP therapy in progressive prostate cancer. Further clinical studies still need to confirm whether this treatment brings survival benefits to patients.

## Data Availability

The original contributions presented in the study are included in the article/Supplementary Material, further inquiries can be directed to the corresponding authors.
